# Metabolic Consequences of Hybridization in European Water Frogs (*Pelophylax esculentus* complex)

**DOI:** 10.1002/ece3.72504

**Published:** 2025-11-16

**Authors:** Adam Hermaniuk, Magdalena Czajkowska, Maciej Pabijan, Wilco C. E. P. Verberk, Marcin Czarnoleski

**Affiliations:** ^1^ Faculty of Biology, Life History Evolution Group, Institute of Environmental Sciences Jagiellonian University Kraków Poland; ^2^ Faculty of Biology University of Białystok Białystok Poland; ^3^ Faculty of Biology, Institute of Zoology and Biomedical Research Jagiellonian University Kraków Poland; ^4^ Department of Animal Ecology and Physiology, Radboud Institute for Biological and Environmental Sciences (RIBES) Radboud University Nijmegen the Netherlands

**Keywords:** amphibians, cell size, introgression, oxygen limitation and temperature, polyploidy, species distribution

## Abstract

Hybridization influences speciation processes, either slowing or reversing species differentiation due to gene flow and recombination, or else accelerating speciation via adaptive introgression and/or polyploidization. One of many consequences of polyploidization is an increase in cell size associated with genome multiplications. Although cell size is regarded as affecting Darwinian fitness across environmental gradients, particularly due to its effects on oxygen transport, fitness effects of changes in cell size associated with hybridization are not well understood. In this study, we examined the effects of ploidy level, our proxy for cell size, and genotypes on metabolic responses to thermal and oxygen conditions in tadpoles of European water frogs (*Pelophylax esculentus* complex). Hybrids ( *P. esculentus* ), originating from the primary hybridization between *P. lessonae* (genotype LL) and *P. ridibundus* (RR), were crossed to produce tadpoles with various genotypes (RR, LR, LLR, LRR) and ploidy levels (diploid, triploid). Our results indicate that triploids, particularly LLR, are most susceptible to oxygen limitation in hypoxic water. Additionally, RR progeny with introgressed *P. lessonae* mtDNA exhibited the lowest metabolic rates under normoxia, suggesting mitochondrial dysfunction due to mitonuclear incompatibility. The greater oxygen limitation in triploids, particularly under hypoxic conditions, may explain their preference for cooler climates. In a time of rapid environmental change, uncovering the physiological trade‐offs associated with hybrid and polyploid genotypes, in connection with cell size changes, is a promising framework for predicting species responses to shifting oxygen and temperature regimes.

## Graphical abstract

Our study links hybridization, ploidy, and cell size to metabolic performance under varying oxygen and temperature conditions in an amphibian larval model. By revealing physiological constraints tied to genotype and mitonuclear mismatch, it provides mechanistic insights into how hybrid and polyploid organisms may respond to environmental change.

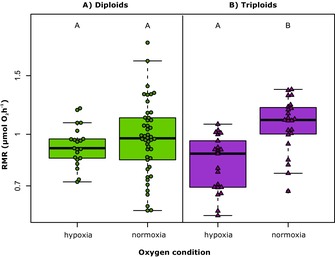

## 1 Introduction

Hybridization has captivated the attention of researchers for decades due to its profound implications for biodiversity, adaptation, and speciation (Brauer et al. 2023). By facilitating the transfer of genes between closely related species, hybridization can result in an increased ability to exploit novel and diverse ecological niches (Abbott et al. 2013; Kulmuni et al. 2023). Indeed, recent genetic evidence suggests that hybridization is a pivotal process in animal evolution including the evolutionary history of our own species (Edelman and Mallet 2021; Fijarczyk et al. 2018).

Species hybridization is frequently associated with polyploidization involving the inheritance of additional sets of chromosomes (Doyle and Coate 2019). Polyploidization can arise through autopolyploidy or allopolyploidy (see Maldung 2013, for a review), with the latter being particularly common in hybrid taxa. In diploid hybrids, the inheritance of divergent parental chromosomes often leads to chromosomal incompatibilities that disrupt meiosis and gametogenesis. These reproductive barriers can, however, be overcome by the formation of allopolyploid lineages, which restore meiotic stability. Under favorable conditions, allopolyploids can benefit from “hybrid vigor,” outperforming their parental species by combining advantageous traits from both lineages. In this way, a newly formed polyploid hybrid can shift its ecological niche avoiding the challenges faced by diploid hybrids associated with chromosome segregation (Mason and Pires 2015; Otto 2007). Indeed, allopolyploidization has been linked to rapid ecological divergence (Coyne and Orr 2004) and adaptation to post‐glacial or arid climates (David 2022).

Polyploidization leads to a number of profound consequences, including the emergence of organisms with larger cells and alterations in genome dosage and gene expression (Doyle and Coate 2019). The increase in cell size, a well‐known phenomenon associated with increased ploidy (Gregory 2001), itself is considered to affect different aspects of organismal performance. Following the theory of optimal cell size (TOCS; Atkinson et al. 2006; Kozłowski et al. 2020; Szarski 1983; Verberk et al. 2021, for a recent synthesis see Czarnoleski and Verberk 2025), a given cellular architecture of the body leads to different fitness consequences across environmental gradients. These effects largely result from cell‐size scaling of transport capacity and the metabolic costs of tissue maintenance. The transport capacity of a cell, including the delivery of oxygen to mitochondria, strongly depends on diffusion distances inside the cell and on the surface area of the plasma membrane available for the exchange of molecules between the cytoplasm and the extracellular environment. The involvement of the plasma membrane in oxygen transport is even more pronounced given that oxygen diffuses more readily through the lipidic environment inside membranes than through aqueous cytosol (Subczynski et al. 1989). This suggests that the nets of plasma membranes in clusters of cells serve as transport pathways that facilitate the penetration of tissue by oxygen. For ectotherms, whose metabolic rates increase with increasing temperatures, TOCS predicts that larger cells, with their smaller surface‐to‐volume ratio, face increased difficulties in meeting oxygen requirements. This can be especially challenging in warm and hypoxic water, where aquatic ectotherms are at increased risk of unbalancing their demand for oxygen. In such conditions, organisms built of smaller cells can enhance oxygen transport into tissues and mitochondria to maintain the balance between the environmental oxygen supply, which increases linearly with temperature, and the metabolic demand, which increases exponentially with temperature (Pörtner 2010; Verberk et al. 2011; Woods 1999).

**FIGURE 1:**
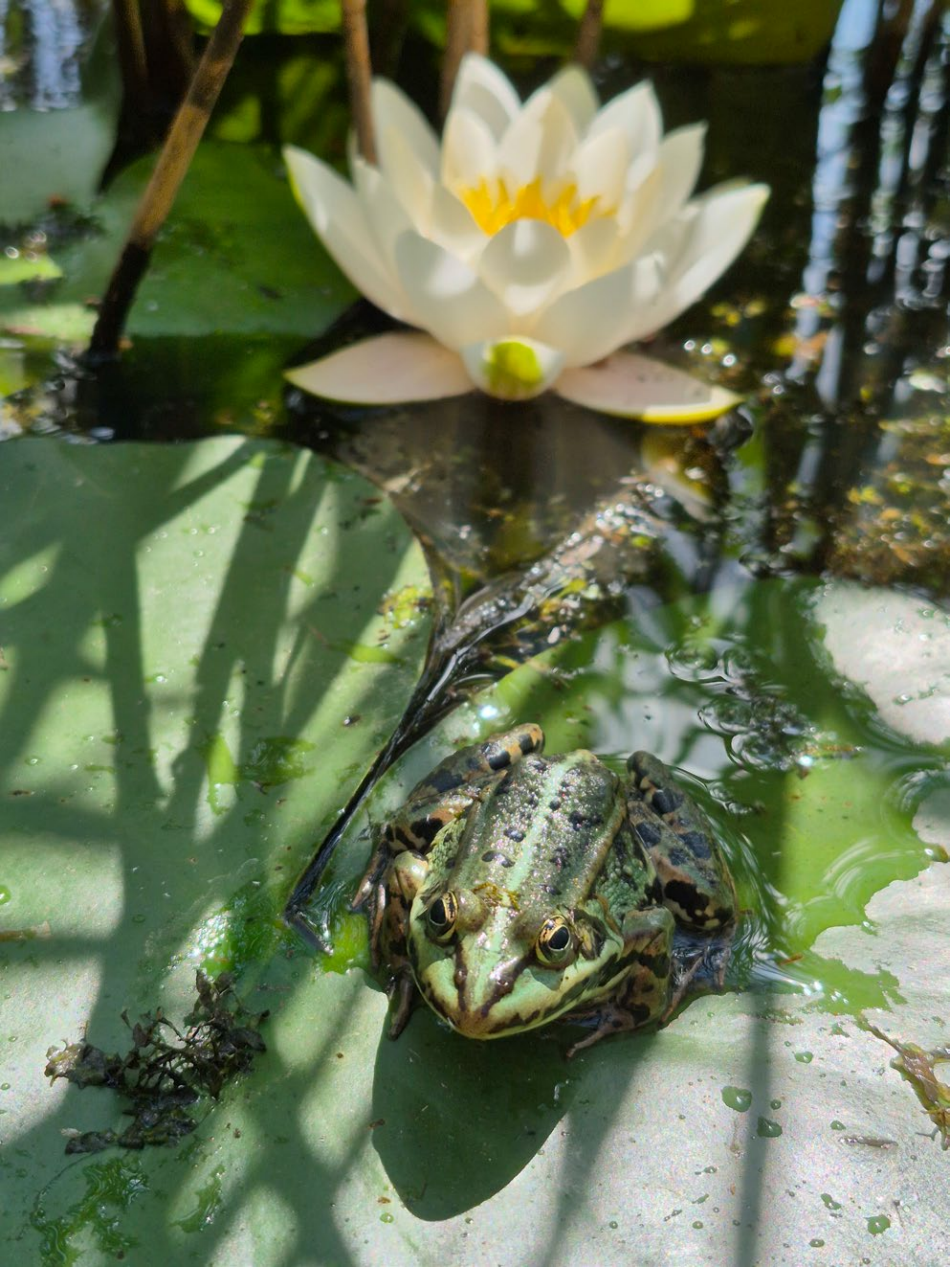
A hybrid edible frog (*P* *elophylax esculentus*) in its natural habitat in the West Pomeranian region, Poland. Photo by Adam Hermaniuk.

**FIGURE 2:**
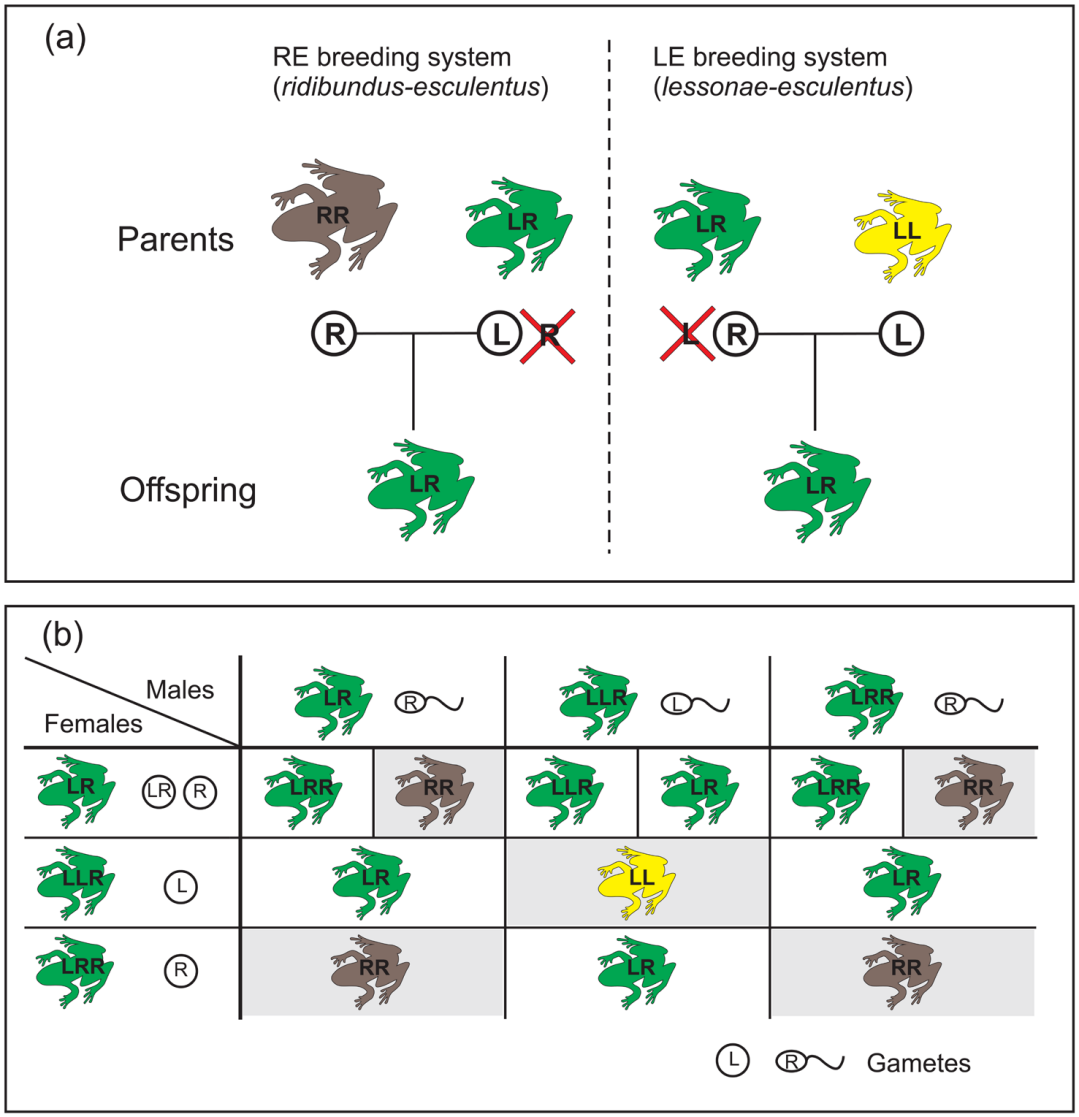
(a) Typical patterns of genome inheritance in hybridogenetic reproduction of the European water frogs (*Pelophylax esculentus* complex) in two breeding systems (RE and LE) involving parental species and hybrids. RR— *P. ridibundus* genotype (brown); LR—hybrid genotype (green); LL— *P. lessonae* genotype (yellow). Circle denotes gametes, and red strikethrough indicates elimination of one of the genomes before meiotic division. (b) Typical reproduction patterns of water frogs (*Pelophylax esculentus* complex, but see Christiansen 2009 for more complex cases) in all‐hybrid populations without the involvement of parental species ( *P. lessonae* and *P. ridibundus* ). As a result of mating between three genotypic forms of hybrids, up to five different genotypes can occur in the offspring, including individuals with genotypes representing the parental species (gray background). Non‐hybrid offspring (LL and RR) that are formed in every generation typically do not reach sexual maturity, although tadpoles of RR genotypes are fully viable (see results of this study).

Our study explores the effects of thermal and oxygen conditions on oxygen consumption in tadpoles of European water frogs (*Pelophylax esculentus* complex, Figure 1) representing different hybridizing genotypes and ploidy levels. We take advantage of a peculiar and complex breeding system of water frogs (Figure 2; for further details see Graf and Polls‐Pelaz 1989) and the availability of breeding laboratory methods for obtaining water frog offspring with specific genotypes (see Table 1 for our breeding results), as well as tight links between ploidy level and cell size in water frog tadpoles. Previous studies have shown that triploids have about 50% more DNA than diploids (Ogielska et al. 2004), which corresponds to larger cell sizes in different cell types, including erythrocytes, epidermal cells, and hepatocytes (Hermaniuk et al. 2016). We hypothesized that triploid tadpoles composed of larger cells compared to diploids, would have a reduced capacity for supplying tissue with oxygen. This would be manifested as a lower oxygen consumption rate at higher water temperatures under hypoxic conditions compared to diploids.

Central European populations of water frogs are typically composed of two diploid parental species—the pool frog, *Pelophylax lessonae* (Camerano, 1882), with genotype LL, and the marsh frog, *Pelophylax ridibundus* (Pallas, 1771), with genotype RR—as well as their diploid hybrid, the edible frog, *Pelophylax esculentus* (Linnaeus, 1758), with genotype LR. The hybrid form is the most widespread water frog taxon in central Europe, and it combines hybrid origin with hemiclonal reproduction (hybridogenesis). This reproductive mode involves the selective transmission of one of the two parental genomes, either R or L, to the next generation. The other genome (L or R respectively) is renewed to form a hybrid through mating with one of the parental species (LL or RR), which serve here as “sexual hosts” (Figure 2a, for review see also Lavanchy and Schwander 2019). In addition to populations with only diploid frogs, all‐hybrid populations can perpetuate without the involvement of parental species (Figure 2b). In these populations, diploid hybrids emerge from the coupling of homospecific genomes in triploids with different combinations of parental genomes (LLR or LRR) (see Christiansen and Reyer 2009 for further details). It is noteworthy that in Europe, multiple variants of the systems described above have been documented, involving different combinations of parental species and both diploid and triploid hybrids (Fedorova et al. 2025; Hoffmann et al. 2015; Rybacki and Berger 2001). For reasons not yet understood, the reproductive systems of frogs are not randomly distributed geographically. For example, all‐hybrid populations with a high percentage of polyploids occur mainly in the northern parts of the distribution range of the species complex (Christiansen and Reyer 2011; Hoffmann et al. 2015). Interestingly, the mtDNA of *P. lessonae* (L‐mtDNA) has undergone multiple introgressions into *P. ridibundus* over a large geographical area, most likely through an intermediate stage of *P. esculentus* . Despite the significant divergence of mtDNA between the two mitochondrial genomes (∼12%, including functional changes in protein‐coding genes, Hofman et al. 2012), *P. ridibundus* with L‐mtDNA are common in some areas, especially in the northern part of the European species range, (Jośko and Pabijan 2021; Litvinchuk et al. 2020; Plötner et al. 2008). This suggests that the introgression of L‐mtDNA into *P. ridibundus* (and *P. esculentus* ) is not profoundly maladaptive. On the other hand, the physiological importance of oxidative phosphorylation, to which mtDNA gene products contribute, means that even minor biochemical inefficiencies through mismatches between mtDNA and nuclear DNA elements can have pronounced fitness consequences (mitonuclear incompatibility; Hill 2019). To account for the effects of potential genomic conflicts involved in mitonuclear interactions, all tadpoles involved in our study were mitotyped. This way, our results help to explore whether genotype × temperature × oxygen interactions in relation to ploidy and cell size can be involved in shaping the spatial distributions of genetic forms of the *P. esculentus* species complex. Such information is needed not only to better understand the environmental forces that have produced the current ecological patterns of water frogs, but also to predict the drivers of ecological shifts in water frog populations associated with transitions in global climate. It should be emphasized that amphibians have been identified as one of the most vulnerable groups of animals to these changes, facing a high risk of extinction (Luedtke et al. 2023; Pottier et al. 2025), and our study addresses the novel idea that these challenges may depend on the ploidy level and cell size of amphibians.

In summary, our study aims to examine how cell size and introgression affect metabolic responses to temperature and oxygen. We hypothesize that triploid tadpoles, with larger cells, have a reduced capacity to supply tissues with oxygen, leading to lower oxygen consumption, particularly under hypoxic conditions. Additionally, we predict that mitonuclear mismatches due to introgression affect oxygen consumption rates.

## 2 Material and Methods

### 2.1 Ethics Statement

All procedures implemented in this study adhered to the principles of animal care and national laws (approvals: DZP‐WG.6401.02.6.2017.dł from The General Directorate for Environmental Protection; 32/2017, from the Local Ethical Committee for Animal Experiments in Olsztyn, Poland).

### 2.2 General Overview of the Procedures

We sampled wild frogs and used them in controlled breeding to produce tadpoles for further analysis. Parental frogs were identified based on morphological traits, ploidy, and genetic markers. After crossing in semi‐natural conditions, fertilized eggs were classified by size to obtain diploid and triploid tadpoles, which were then reared under controlled laboratory conditions. Then their metabolic rates were measured under different temperature and oxygen regimes. Following metabolic trials all tadpoles were weighed, genotyped, and mitotyped.

### 2.3 Parental Frogs and Crossing

Frogs used in this study were collected from all‐hybrid populations (West Pomeranian Voivodeship, Poland) where diploid (LR) coexist with triploid (LLR and LRR) hybrids. Frogs were caught in four water bodies shortly before the breeding season (early May), usually at night by hand or during the day with a net. All animals were sexed by the presence (males) or absence (females) of nuptial pads on thumbs and vocal sac openings. Taxonomic identification, ploidy and genome composition of all specimens were preliminarily determined on the basis of morphological indexes and erythrocyte size (Kierzkowski et al. 2011) and then confirmed using microsatellites (described below). Fingertips were clipped to obtain blood smears and the biopsies were used in further molecular analyses. All collected frogs were transported to the faculty animal facility, where they were kept in a climate‐controlled chamber at 22°C under a natural photoperiod. The frogs were kept in terraria (84 × 50 × 40 cm; maximum 10 frogs per terrarium), covered with mesh to provide ventilation. Artificial light above the terraria allowed the frogs to bask. Each terrarium was equipped with a litter tray and a terrestrial habitat. The animals were fed crickets.

To produce tadpoles for the metabolic measurements, we crossed 14 pairs of adult frogs (Table 1) out of 49 frogs collected in the field. We used a noninvasive crossing procedure following the protocol of Berger (1988) with some modifications described below. Parental pairs (crosses) were bred in seminatural conditions (outdoors) in tanks (84 cm × 50 cm × 40 cm) covered with mesh. Tanks were placed in a sunlit area, filled with pond water up to ¼ height and supplemented with plants. Usually within a few days the frogs spawned in the tanks. If spawn did not appear within a few days, we changed the male in a given cross. After spawning, each clutch was immediately transferred to the laboratory and fertilized eggs were divided into separate size classes. Females of *P. esculentus* produce gametes of various sizes that can be easily divided into classes (small, medium, and large) associated with different ploidy levels (Berger 1988; Czarniewska et al. 2011). To obtain diploid offspring, diploid or triploid males were crossed with triploid females, either LLR or LRR, producing mostly haploid L or R gametes, respectively. Only eggs classified as small or medium (haploid gametes in most cases) were taken after fertilization for further rearing. A similar procedure was applied to obtain triploid offspring, with the exception that diploid females LR, producing a high percentage of diploid gametes, were used and large eggs (diploid LR gametes in most cases) were taken for further rearing except for two crosses (18A and 21A; see Table 1 for further details) in which high mortality of embryos originating from large eggs was observed. After the crossing procedure all adult frogs were released to their sites of capture.

**TABLE 1:** Mating design of frogs of *Pelophylax esculentus* complex used to produce tadpoles for metabolic measurements in this study.

Cross ID	Female genotype	Male genotype	Egg size selected for tadpole rearing	Tadpole genotype	
				Expected	Observed
1a	LR	LLR	Large	LLR	LLR
2a	LR	LR	Large	LRR	LLR
3a	LRR	LR	Not selected	**RR**	**RR**
4c	LRR	LLR	Small	LR	LR
6b	LR	LLR	Large	LLR	LLR
10a	LRR	LLR	Small	LR	LR
12b	LR	LRR	Large	LRR	LRR
13b	LR	LLR	Large	LLR	LLR
16b	LLR	LR	Medium	LR	LLR, LR
17a	LLR	LR	Medium	LR	LLR, LR
18a	LR	LR	Small	**RR**	**RR**
19a	LR	LR	Large	LRR	LRR
21a(S)^a^	LR	LR	Small	**RR**	**RR**
21a(L)^a^	LR	LR	Large	LRR	LR
22a	LLRR	LR	Not selected	**(?)** ^b^	**RR**

*Note:* To obtain a high percentage of diploid or triploid progeny, specific size classes of fertilized eggs were pre‐selected for offspring rearing, as predictors of tadpole genotypes (expected). The tadpole genotypes were later verified with microsatellite methods (observed). Some females produced eggs of one size only; in such cases, egg size was not considered in tadpole rearing (“Not selected”). *Pelophylax ridibundus* (RR) individuals were labeled in bold—they do not occur among adults in the field, but apparently offspring with this genotype are produced. For details of the genetic breeding system within *P. esculentus* complex frogs, see Figure 2.

^a^ Tadpoles from cross 21a were reared in two separate tanks depending on the size of the eggs (S—small, L—large) they developed from.

^b^ The reproductive pattern of tetraploid individuals has been rarely documented (see Discussion for details).

### 2.4 Rearing Tadpoles

The larvae were reared in a climatic chamber at 20°C (±0.5°C) under a 12 h:12 h light:dark photoperiod throughout their development. Developing embryos and hatchlings from each cross were kept in separate plastic litter trays filled with tap water at 20°C until tadpoles reached developmental stage 25 (free swimming and independently feeding larvae) according to Gosner (1960). After reaching this stage, groups of 50 healthy‐looking tadpoles were selected and transferred to aquaria (50 cm × 35 cm × 30 cm), maintaining the same arrangement of containers as before. At each stage water was continuously aerated using air stones for embryos and hatchlings, and air‐driven filters (PZC 300, Aquael) for tadpoles, which also removed food remnants and feces. Litter trays and tanks were inspected daily, and dead larvae were removed. At least three times a week, half of the water from each tank and litter tray was replaced with aged tap water stored for at least 24 h at 20°C. Tadpoles were fed *ad libitum* two times per day with a powder mix consisting of one part dried nettle and one part commercial fish food (Supervit, Tropical). All tadpoles that were not selected for further experiments (see below) were anesthetized with a 0.25% solution of MS 222 (Sigma‐Aldrich) in water.

### 2.5 Metabolic Measurements of Tadpoles

The experimental design aimed to measure metabolic rates in tadpoles representing different genotypes and ploidy levels raised at the same developmental temperature (20°C). Based on published evidence for water frog tadpoles (Hermaniuk et al. 2016) and a common approach in other organisms (Gregory 2001; Miettinen et al. 2017), we considered ploidy level as a proxy of systemic trends in cell size. During the measurements, tadpoles experienced either developmental temperature (20°C) or increased temperature (24°C), combined with two oxygen conditions in the water: normoxia (average initial level 7.22 mg O_2_/L) and hypoxia (average initial level 2.72 mg O_2_/L).

The metabolic measurement assays were conducted on tadpoles without visible morphological abnormalities, which reached a medium Gosner developmental stage of 30 (range: 26–35), a stage when tadpoles primarily rely on oxygen dissolved in water (Czopek 1965). Two days prior to the measurements, all tested animals were deprived of food. The assays measured the oxygen consumption of individual tadpoles in a closed respirometry system equipped with an oxygen electrode and a temperature sensor (Yellow Spring Instruments, Yellow Spring, OH), connected to a CyberScan PCD 6500 bench meter (Eutech Instruments, Singapore). Each metabolic assay (normoxia and hypoxia) involved two tadpoles measured simultaneously in hermetically sealed glass chambers completely filled with water, along with a reference chamber without a tadpole (blank). The normoxic and hypoxic conditions inside chambers (including the blank) were achieved by bubbling the water with gases: either ambient air for normoxia using an air pump or nitrogen for hypoxia supplied from a high‐pressure gas cylinder. After setting water conditions, all chambers were submerged in a water bath equipped with a cooling/heating system, ensuring precise temperature stabilization (±0.1°C). A small automatic stirrer, determined not to disturb the tadpoles, maintained water mixing within each chamber.

Before each metabolic assay, all chambers were sterilized using 70% EtOH to prevent microbial accumulation over time. The assay lasted at least 4 h, including a 30‐min acclimation period, which was excluded from data analysis. Oxygen consumption was recorded every 5 min with automatic temperature and pressure corrections applied. Measurement durations were set to ensure that the oxygen level in the chamber did not drop below 70%–80% of the initial value. Over time, the oxygen level in the chamber decreased due to oxygen uptake by the tadpoles and the activity of an unidentified microbial community. Tadpoles were allowed spontaneous movement during the assays, so that our measurements reflect both energy requirements for maintenance and movement, which conforms to the definition of routine metabolic rates (RMR, for reference see Halsey et al. 2015). RMR was calculated from the formula RMR = *aV*, where the coefficient *a* is the slope in a linear regression of dissolved oxygen concentration versus time (μmol/L/h) and *V* is the volume of the metabolic chamber (L). The linear regressions provided a good fit for each of the RMR traces (average *R* ^2^ = 0.98). Additionally, in hypoxic conditions, oxygen slowly leaked into the chamber from the surrounding environment (ingress). The metabolic rate of each tadpole was adjusted based on the oxygen readings in the reference blank chamber, to account for the effects of microbial activity in normoxia and oxygen ingress in hypoxia. Note that tadpole oxygen consumption rates were ~4 times greater than the average rates for background respiration and the average rates for oxygen ingress (background: 19.4%; ingress: 23.1%).

In total, metabolic data were successfully collected for 111 individuals from 14 crosses (Table 1). After the metabolic assay, tadpoles were placed on blotting paper to remove excess water before weighing them to the nearest 0.1 mg. Then they were anesthetized with a 0.25% solution of MS 222 (Sigma) in water and stored at −20°C in plastic tubes for genotyping and mitotyping.

### 2.6 Molecular Analyses

The DNA of parental frogs was extracted from blood smears prepared on microscopic slides. Dry blood samples were collected from the slides using swabs soaked in PBS buffer (phosphate‐buffered saline) and processed with the QIAamp DNA Micro Kit (Qiagen) according to the manufacturer's protocol for extracting genomic DNA from dried blood spots. The DNA was then eluted in 50 μL of AE buffer. Tadpole DNA was extracted from small fragments of tails using the Genomic Mini Kit (A&A Biotechnology), following the manufacturer's protocol, and eluted in 120 μL of Tris buffer. Parental frogs and tadpoles were genotyped by fragment length analysis as described in detail by Hermaniuk et al. (2020). Briefly, a set of four pairs of microsatellite primers were applied: loci (Cala27 and RlCA18) PCR‐amplify exclusively in *P. lessonae* (Garner et al. 2000); amplification of the other two (Res22 and Rrid169A) is restricted to *P. ridibundus* (Hotz et al. 2001; Zeisset et al. 2000). Diploid hybrids (LR) amplify one allele at each of the four loci. LLR triploids amplify one allele at both loci specified by the *P. ridibundus* genotype and two alleles at least at one locus specified by the *P. lessonae* genotypes, and inversely in LRR triploid genotypes (one allele at the L‐specific loci and two alleles at least at one R‐specific locus). These four markers are among the most polymorphic loci tested so far and precisely identify *ridibundus* and *lessonae* genotypes (Czernicka 2013).

Mitotyping of tadpoles was done using a PCR‐based method developed in Jośko and Pabijan (2021). Briefly, two primer pairs that selectively amplify either mtDNA of *P. lessonae* (LESF1/LESR1) or *P. ridibundus* (RIDF1/RIDR1) were used to mitotype all progeny. An individual was designated as containing *P. lessonae* mtDNA if PCR with the primer pair LESF1/R1 revealed a clear band of expected size on agarose, but PCR with RIDF1/R1 failed, and conversely, an individual was considered as having *P. ridibundus* mtDNA if PCR with the former primer pair failed but was successful with the latter. Positive and negative controls were used in each reaction.

### 2.7 Statistical Analysis

Statistical analysis was conducted in R version 4.2.1 (R Core Team 2022; https://www.R‐project.org/). The *lmer* function from the *lme4* package (Bates et al. 2015) was used to fit linear mixed effects models (LMM). For significant categorical predictors that had multiple levels, we reran models to assess pair‐wise comparisons, by specifying the category of interest as the model intercepts to create the desired contrasts. The *visreg* (Breheny and Burchett 2017) and *beeswarm* packages (Eklund and Trimble 2021) were used to visualize results. Data on RMR and body mass were log_10_‐transformed prior to the analysis to meet assumptions of linearity.

To study the effects of ploidy (our proxy of cell size) on RMR, we used LMMs, with body mass, test temperature (20°C and 24°C) and test oxygen conditions (normoxia and hypoxia), and ploidy level (2n or 3n) as fixed effect factors, and the cross (i.e., 14 pairs of parents) as the random factor. In this analysis, data on different genotypes were pooled within each ploidy. In all analyzed LMMs, the random factor did not significantly change the slopes of linear regressions. We ran a series of models to test whether including interactions between ploidy, oxygen, and temperature yielded a model that was better supported (lower AIC) compared to a model that only included the main effects. To study the effects of genotype on RMR, we used another LMM with a similar procedure of selecting the best‐fitting model as outlined above, but these models considered genotype (LR, RR, LLR, and LRR) as a fixed factor and information on ploidy level was omitted.

## 3 Results

### 3.1 Genome Composition of Parental Frogs and Progeny

All 49 adult frogs collected in the field (25 females, 24 males) were identified as *P. esculentus* . Our microsatellite analysis showed that among the collected frogs, 31 were diploid (LR), and 17 were triploid (8 LLR, 9 LRR). Unexpectedly, we also identified one individual (cross 22A) as a tetraploid (LLRR) and this individual was a female that produced R gametes exclusively (see Appendix 1, Table A1 with detailed results of genotyping).

The progeny of eight parental pairs selected for the crossing procedure represented three *P. esculentus* genotypes: LR, LLR, and LRR. In two crosses (16B and 17A), we expected only LR offspring based on egg size, but observed progeny with two ploidy levels (Table 1), against predicted patterns of genome inheritance in these females (see Figure 2b). In four parental pairs, we obtained RR offspring, representing the genotype of one of the parental species, *P. ridibundus* (Table 1, see also Appendix 1, Table A1 for a detailed summary of the genotype composition for all individuals in each cross). Our analysis of mtDNA showed that all examined progeny carried only *P. lessonae* mitochondrial DNA, including tadpoles identified as non‐hybrid *P. ridibundus* .

### 3.2 Routine Metabolic Rate (RMR)

**FIGURE 3:**
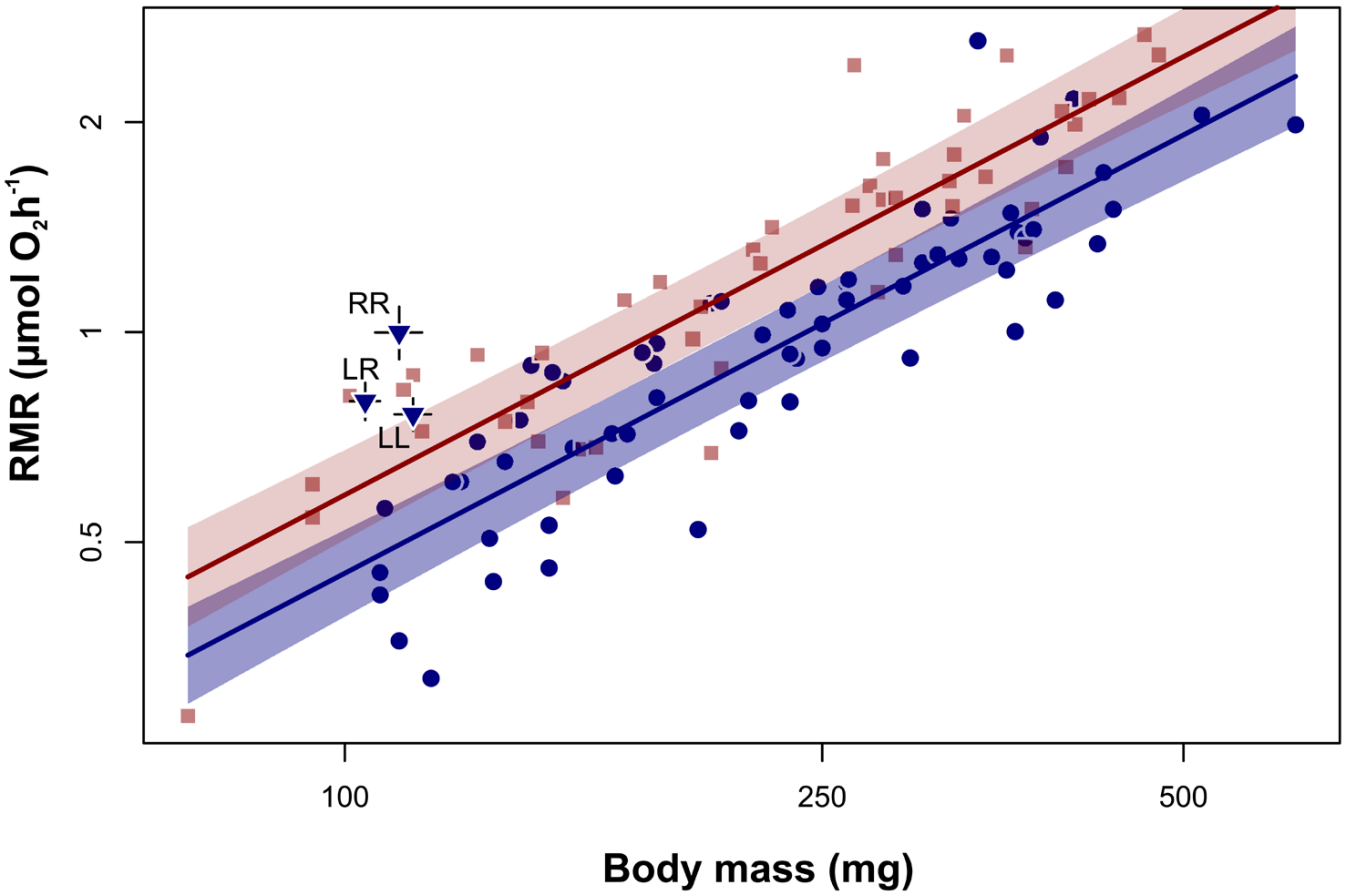
Routine metabolic rate (RMR per individual) in tadpoles within *Pelophylax esculentus* complex under different test temperatures (red—24°C, blue—20°C). Values of RMR are model‐based ‐partial residuals, for details of the statistical model refer to Table 3. The triangles indicate the average oxygen consumption rates (± SE) under normoxia at 20°C for *P. lessonae* (LL), *P. esculentus* (LR), and *P. ridibundus* (RR) from a previous study (Plénet, Hervant, and Joly 2000).

**FIGURE 4:**
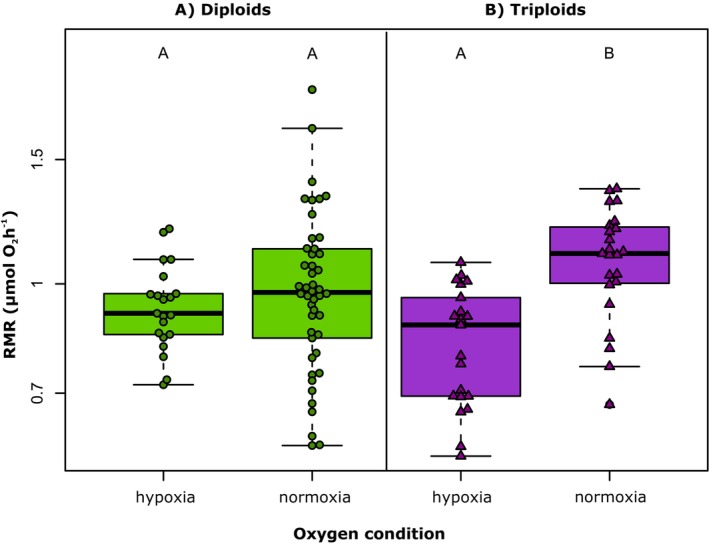
Routine metabolic rate (RMR per individual) under different oxygen conditions in diploid (panel A) and triploid (panel B) tadpoles of the *Pelophylax esculentus* complex, based on a linear mixed effects model reported in Table 3. Values of RMR are partial residuals, without the effects of other predictors (temperature and body mass). Different genotypes were pooled within each ploidy category for the analysis. Letters indicate statistical significance in pairwise comparisons (*p* < 0.05), estimated after rerunning the main model with specific groups in model intercepts that allowed desired contrasts for comparisons. Central tendencies in groups are indicated by a median value with other quantiles.

**FIGURE 5:**
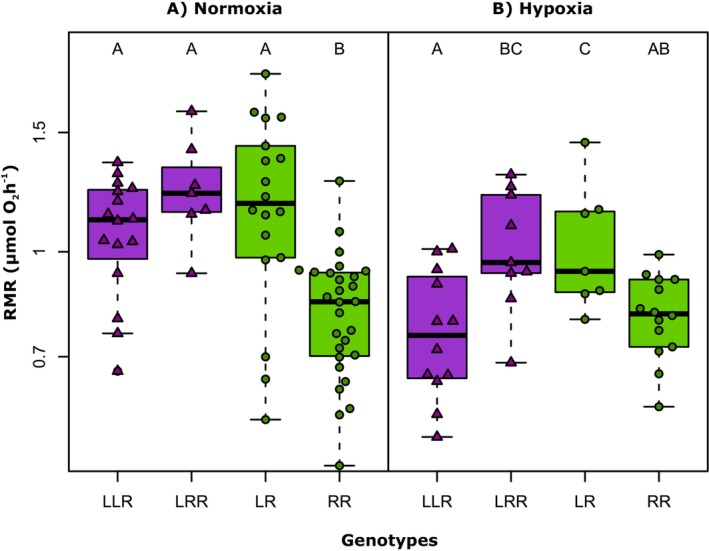
Routine metabolic rate (RMR per individual) under normoxia (panel A) and hypoxia (panel B) in different genotypes of tadpoles of the *Pelophylax esculentus* complex, based on a linear mixed effects model reported in Table 4. Values of RMR are partial residuals, without the effects of other predictors (temperature and body mass). The green color represents diploids, and the purple color represents triploids. Letters indicate statistical significance in pairwise comparisons (*p* < 0.05), estimated after rerunning the main model with specific groups in model intercepts that allowed desired contrasts for comparisons. Central tendencies in groups are indicated by a median value with other quantiles.

Our selection procedures (Table 2) of models, either in the analysis of RMR in relation to ploidy, or in relation to genotype, resulted in a similar model structure. The strongest support in data received models that included all main predictors, namely body mass, temperature and oxygen availability, and an interaction between oxygen availability and either ploidy or genotype. The best‐fitting models were further used to analyze statistical significance and effect size of model predictors. In each model, effects of body mass and temperature explained a large part of the observed variation in RMR (~70%). Therefore, results of models were visualized using partial residual plots (Figures 3, 4, 5).

**TABLE 2:** Results of model selection to explain variation in tadpole oxygen consumption rates (routine metabolic rate, RMR). The first set of models considered the effect of ploidy (2n vs. 3n) and the second set the effect of the genotype (LR vs. RR vs. LLR vs. LRR).

	*k*	AICc	ΔAICc	*w*_*i*_	*LL*
Effects of ploidy					
RMR ~ M + T + O_2_ + Ploidy	7	−167.08	3.61	0.11	91.08
RMR ~ M + T × O_2_ + Ploidy	8	−165.97	4.73	0.07	91.69
RMR ~ M + O_2_ + T × Ploidy	8	−164.80	5.89	0.04	91.10
RMR ~ M + T + O_2_ × Ploidy	**8**	**−170.69**	**0**	**0.70**	**94.05**
RMR ~ M + T × O_2_ × Ploidy	11	−166.43	4.26	0.08	95.55
Effects of genome					
RMR ~ M + T + O_2_ + Genotype	9	−181.23	1.94	0.23	100.50
RMR ~ M + T × O_2_ + Genotype	10	−180.36	2.80	0.15	101.28
RMR ~ M + O_2_ + T × Genotype	12	−175.31	7.86	0.01	101.24
RMR ~ M + T + O_2_ × Genotype	**12**	**−183.17**	**0**	**0.61**	**105.17**
RMR ~ M + T × O_2_ × Genotype	19	−171.79	11.37	0.00	109.07

*Note:* Models included log body mass (M), test temperature (T: 20°C and 24°C), test oxygen level (O_2_: normoxia and hypoxia) as fixed factors. Values for Mass and RMR were log_10_ transformed prior to analyses. All models include parental cross as a random effect on the intercept. The number of parameters (*k*), the corrected Akaike's information criterion (AICc), the difference in AICc value concerning the best model ΔAICc, Akaike's weights (*w*_*i*_) and log‐likelihood (*LL*) are indicated for each model. Bold indicates models best supported by data. These models, one for ploidy effects (Table 3) and one for genotype effects (Table 4) were further analyzed for statistical significance and effect size of predictors.

### 3.3 Effects of Ploidy

The best‐fitting model for ploidy effects showed that oxygen consumption increased with tadpole mass and test temperature (Figure 3, Table 3). The mass‐scaling exponent was estimated to be 0.90. The effects of temperature indicated the quotient Q_10_ equal to 1.91. Hypoxic conditions significantly reduced oxygen consumption rates in triploid (3n) tadpoles, whereas no such reduction was observed in diploid (2n) individuals (Figure 4). This was reflected in a significant interaction between ploidy and oxygen condition, indicating that triploids were more sensitive to oxygen limitation than diploids (Table 3).

**TABLE 3:** Results of a linear mixed‐effects model (LMM) for the routine metabolic rates (RMR) in tadpoles of the *Pelophylax esculentus* complex with different ploidy levels (2n vs. 3n).

Factor	Coefficient	SE	df	*t*	*P*
Intercept	−0.029	0.109	106.215	−0.268	0.790
M	0.899	0.050	109.273	17.780	< 0.001
T	0.028	0.005	98.649	6.051	< 0.001
O_2_	0.021	0.026	100.311	0.812	0.4188
Ploidy	−0.055	0.042	46.833	−1.296	0.201
O_2_ × Ploidy	0.097	0.039	100.066	2.477	0.015

*Note:* The model represents an LMM best supported by data (see Table 2) and its results are visualized in Figures 3 and 4. The model included log body mass (M), test temperature (T: 20°C and 24°C), test oxygen level (O_2_: normoxia and hypoxia) as fixed factors. The intercept represents the expected value for diploids under hypoxia.

### 3.4 Effects of Genotype

The best‐fitting model for genotype effects indicated an increase in oxygen consumption with tadpole mass and test temperature, similarly to the results of the model for ploidy levels. The scaling exponent for body mass was estimated at 0.88, while the effects of temperature indicated Q_10_ equal to 1.91. Generally, hypoxic conditions reduced oxygen consumption rates in tadpoles, and the overall pattern of differences in RMR between different genotypes was similar under normoxia and under hypoxia. However, a significant genotype × oxygen conditions interaction (Table 4, Figure 5) indicates that quantitative responses of different genotypes to oxygen conditions were significantly different. In particular, the most pronounced reduction in oxygen consumption under hypoxia was observed in LLR, followed by LRR. In contrast, genotypes LR and RR exhibited only a slight tendency for reduced RMR at hypoxia. Noteworthy, under normoxic conditions, RR individuals, which represented tadpoles with introgressed L‐mtDNA, had the lowest oxygen consumption rates among all studied genotypes (Figure 5, panel A).

**TABLE 4:** Results of a linear mixed‐effects model (LMM) for the routine metabolic rates (RMR) in tadpoles of *Pelophylax esculentus* complex with different genotypes (LR, RR, LLR, LLR).

Factor	Coefficient	SE	df	*t*	*P*
Intercept	−0.129	0.104	111	−1.237	0.219
M	0.885	0.047	111	18.708	< 0.001
T	0.028	0.004	111	6.193	< 0.001
O_2_	0.147	0.036	111	4.073	< 0.001
LR	0.128	0.045	111	2.877	0.005
LRR	0.126	0.043	111	2.936	0.004
RR	0.027	0.037	111	0.724	0.470
O_2_ × LR	−0.093	0.055	111	−1.675	0.097
O_2_ × LRR	−0.060	0.060	111	−0.999	0.320
O_2_ × RR	−0.145	0.047	111	−3.075	0.003

*Note:* The model represents an LMM best supported by data (see Table 2) and its results are visualized in Figure 5. The model included log body mass (M), test temperature (T: 20°C and 24°C), test oxygen level (O_2_: normoxia and hypoxia) as fixed factors. The intercept represents the expected value for LLR individuals under hypoxia.

## 4 Discussion

### 4.1 Oxygen Consumption Rates and Sensitivity to Hypoxia

Our results for European water frog tadpoles indicate that their routine metabolic rates changed almost linearly with body mass (with a scaling exponent of 0.9) and changed by nearly twofold over a 10°C change in ambient temperature. This indicates patterns typical for ectotherms with a pelagic life style at early postembryonic stages of development. For example, pelagic invertebrates generally exhibit steeper mass‐scaling of metabolic rates, with scaling exponents closer to 1 (isometry), compared to their benthic counterparts (Glazier 2022). Ectotherms also typically show isometric metabolic mass‐scaling at a larval stage, and shallower mass‐scaling at the adult stage (Glazier 2022).

We found differences in routine metabolic rates between tadpoles that were associated with ploidy level, genotype and oxygen availability in the environment. Polyploidy is typically associated with increased cell size (Doyle and Coate 2019; Gregory 2001; Miettinen et al. 2017; Van de Pol et al. 2020), which has also been shown for tadpoles representing our study system (Hermaniuk et al. 2016). Therefore, our results strongly suggest significant effects of cell size differences between tadpoles on their physiological performance. All else being equal, diploid organisms should possess a greater number of smaller cells across various tissues compared to polyploid organisms. Consequently, a higher number of smaller cells in diploid vertebrates provides a greater total surface area for diffusive oxygen uptake and transport to mitochondria.

In line with the theory of optimal cell size (TOCS; Czarnoleski and Verberk 2025), we observed a stronger suppression of metabolic rates under hypoxic conditions in triploid tadpoles compared to their diploid counterparts. This response pattern indicates a lower capacity for oxygen uptake by large‐celled organisms, which becomes especially apparent when oxygen availability is limited. Reduced hypoxia tolerance in triploids has been documented to varying degrees in several fish species, including rainbow trout (Benfey and Devlin 2018; Scott et al. 2015), Atlantic salmon (Sambraus et al. 2018), zebrafish larvae (Hermaniuk et al. 2021), and brook char (Jensen and Benfey 2022). The lower tolerance of polyploid fish to hypoxia may also help explain their generally lower aerobic scope (Bernier et al. 2004; O'Donnell et al. 2017) and reduced swimming performance under standard thermal conditions (Van de Pol et al. 2020). Similarly, triploid Atlantic salmon were less tolerant to high seawater temperature and low oxygen saturation, evidenced by lower survival, reduced growth and food intake, and altered swimming behavior (Hansen et al. 2015; Sambraus et al. 2017). Beyond examples of polyploid vertebrates, higher hypoxia sensitivity in large‐celled organisms has also been reported in fruit flies, even though insects have a highly effective tracheal oxygen transport system (Leiva et al. 2023; Szlachcic and Czarnoleski 2021). Furthermore, hypoxia tolerance has been linked to variation in genome size across many different fish species (Verberk et al. 2022). Altogether, these findings emphasize the role of genome/cell size in gas exchange, even in organisms that do not rely on erythrocytes for oxygen transport.

Oxygen limitation should be especially prevalent at higher temperatures, when demand for oxygen is greater (Leiva et al. 2023; Rubalcaba et al. 2020; Szlachcic and Czarnoleski 2021). We found strong effects of temperature and body size on the rates of oxygen consumption, which are generally consistent with former studies involving ectotherms (Glazier 2020; Leiva et al. 2019; Li et al. 2020; Rubalcaba et al. 2020). However, we did not find evidence that polyploid tadpoles showed stronger thermal sensitivity of metabolic rates, nor that thermal sensitivity of tadpoles depended on oxygen conditions. Intraspecific differences in thermal sensitivity of metabolic rate between diploids and triploids have been reported in fish and, more recently, in oysters (Atkins and Benfey 2008; Hermaniuk et al. 2021; Sun et al. 2025). A potential key difference between previous studies and the present study is that our frog system includes multiple genotypes. It is telling that we found greater support for our data on metabolic rates in a statistical model that considered genotypes compared to a very similar model that considered ploidy instead of genotype. Thus, while variation in metabolic responses was adequately captured by ploidy and the interaction between ploidy and oxygen, we found evidence that variation in the rates of oxygen consumption between tadpoles can be additionally explained by differences between genotypes. Note that metabolic differences between ploidy levels or genotypes may be at least partially linked to differences in cell size, as LL individuals have larger cells than RR (Kierzkowski et al. 2011). Thus, LLR may have larger cells than LRR individuals due to a genome dosage effect on cell size and this would match the increased sensitivity to hypoxia observed in LLR tadpoles. Exploring the postulated effects of cell size variation within triploids and within diploids in more detail is a valuable avenue for future studies.

Beyond alterations in ploidy level, hybridization can also be accompanied by other genetic phenomena, such as the introgression of mtDNA from one species into another. This type of introgression results in the co‐occurrence of mitochondrial and nuclear genes, originating from two different species. This is exactly what we have discovered in some of our experimental tadpoles. In particular, we found that *P. ridibundus* (RR genotype) tadpoles have obtained mtDNA from *P. lessonae* (LL genotype). Notably, these introgressed *P. ridibundus* tadpoles exhibited a significantly lower metabolic rate under normoxic (i.e., non‐demanding) conditions, when compared to other genotypes. This contrasts with data reported in the literature, which shows that under the same experimental conditions (i.e., normoxic conditions at 20°C), RR tadpoles exhibited the highest rates of O_2_ consumption, when compared to LL and LR tadpoles (Plénet, Hervant, and Joly 2000; see our Figure 3). It is likely that the reduced metabolism that we observed in the introgressed RR tadpoles reflects genomic conflicts and a mismatch between the effects of nuclear and mitochondrial DNAs. Introgression of foreign mtDNA leads to mitonuclear mismatches that can adversely affect cellular respiration and mitochondrial function (Burton and Barreto 2012; Ellison and Burton 2008; Hill 2019). On the other hand, introgressed RR frogs are common in some populations across the northern part of the range of the *P. esculentus* complex (Jośko and Pabijan 2021; Litvinchuk et al. 2020; Plötner et al. 2008). Alternatively, the lower metabolic rate of RR tadpoles in our study could be explained by the coupling of the same R hemiclone in non‐hybrid offspring. In this case, the lower metabolic capacity may be due to the expression of non‐masked deleterious mutations on the clonally transmitted R nuclear genome (Guex et al. 2002). This possibility is supported by the absence of RR frogs in the adult cohort of all‐hybrid populations (Christiansen et al. 2005; Dedukh et al. 2022; Rybacki and Berger 2001; Som and Reyer 2006). However, we note that our non‐hybrid offspring were fully viable at least at the tadpole stage, highlighting the need for further investigation of this phenomenon.

### 4.2 Ecological Implications

It has been repeatedly observed that polyploids perform better in cold conditions than diploids (Dufresne and Hebert 1998; Hansen et al. 2015; Hermaniuk et al. 2016; Pecl et al. 2024; Sambraus et al. 2017; Van de Pol et al. 2021), which is generally consistent with their predominant distribution at higher latitudes (David 2022). It seems that polyploids are at a selective advantage in cold environments; however, the causal mechanisms are still largely unexplored. Predictions of TOCS offer a promising framework to explain the nonrandom geographic distribution of polyploids in light of the links between cell size and physiological performance across different environments (Czarnoleski and Verberk 2025; Glazier 2022; Kozłowski et al. 2020; Miettinen et al. 2017; Verberk et al. 2022). Larger cells reduce the total plasma membrane area, lowering the energetic costs of maintaining membrane function, including composition, fluidity, and ion transport. Consequently, for ectotherms exposed to cold conditions, this can be advantageous by reducing energy demands and thus maintenance costs. Notably, larger cells—particularly those with multiplied genomes—may support biosynthesis in the cold by providing a more favorable intracellular environment for the fast production of key enzymes (Czarnoleski and Verberk 2025; Hessen et al. 2013; Xia 1995).

European water frogs are widely distributed across Europe (Dufresnes et al. 2024), where they naturally encounter a broad gradient of thermal and oxygen conditions in their aquatic habitats (Jakob et al. 2010; Plénet et al. 1998). Previous studies indicate that genotype–temperature interactions influence the larval performance of European water frogs (Hermaniuk et al. 2016; Pruvost, Hollinger, and Reyer 2013). Our data suggest that the degree of tolerance toward hypoxia depends upon tadpole genotype composition through the modulating effect of cell size. Given that tadpoles inhabit small, shallow ponds, hypoxia tolerance, along with temperature, may be key factors influencing their biological processes, including survival rates, time to metamorphosis, and age at metamorphosis. Within this species complex, oxygen availability has previously been shown to differentially influence tadpole growth and development, with hybrid tadpoles displaying intermediate responses to constant hypoxia (Plénet, Pagano, et al. 2000). In our study, we found that triploid tadpoles, particularly those with the LLR genotype, are the most susceptible to oxygen limitation in the water. Based on these results, we could expect a higher frequency of such genotypes in cooler ponds with higher oxygen content. Thus, our results suggest that the interaction between genotype/ploidy level/cell size and oxygen availability may be involved in the spatial distribution of different genetic forms within the *P. esculentus* species complex, at least during the larval stage. A promising avenue for future research is to quantify in detail the temperature–oxygen conditions at individual ponds along climatic clines and test for a relation between these field conditions and the proportions of the different genotypes (LR, LLR, and LRR) of the tadpoles inhabiting these ponds.

It is worth highlighting that we have found interesting aberrations against typical patterns of genome inheritance in the study system (compare Figure 2b with Table 1). The results of our crossing experiments, particularly the successful mating of a tetraploid female, suggest that the reproductive patterns of European water frogs in all‐hybrid populations may be more complex than previously assumed, as also suggested by the results of Pruvost, Hoffmann, and Reyer (2013). Although the presence of tetraploids in populations of the *P. esculentus* complex has been rarely reported (e.g., Borkin et al. 2004; Jakob et al. 2010; Pruvost et al. 2015), models have predicted their higher frequency, especially in all‐hybrid populations (e.g., Christiansen 2009).

In conclusion, our study highlights how cell size and genomic interactions can influence metabolic performance in European water frog hybrids. Lower hypoxia tolerance in triploids with larger cells can help explain their vulnerability to stressors such as hypoxia and heat, and responses to these stressors may shape the distribution of species including polyploids across thermal clines.

## Author Contributions

**Adam Hermaniuk:** conceptualization (equal), data curation (equal), formal analysis (equal), investigation (lead), methodology (equal), project administration (lead), resources (lead), visualization (equal), writing – original draft (lead), writing – review and editing (equal). **Magdalena Czajkowska:** investigation (supporting), resources (supporting), writing – review and editing (supporting). **Maciej Pabijan:** investigation (supporting), resources (supporting), writing – review and editing (supporting). **Wilco C. E. P. Verberk:** conceptualization (equal), data curation (equal), formal analysis (equal), methodology (equal), visualization (equal), writing – original draft (supporting), writing – review and editing (equal). **Marcin Czarnoleski:** conceptualization (equal), methodology (equal), writing – original draft (supporting), writing – review and editing (equal).

## Conflicts of Interest

The authors declare no conflicts of interest.

## Data Availability

The data that support the findings of this study are openly available in Zenodo at https://doi.org/10.5281/zenodo.14672667, (Hermaniuk and Verberk 2025).

## Appendix 1

**TABLE A1:** Genotypes in the *Pelophylax esculentus* complex estimated from the analysis of four microsatellite loci. The loci include two markers that amplify either only in the L genome (L‐specific alleles) or only in the R genome (R‐specific alleles). The analysis was performed on parental frogs and their offspring, representing different family/cross groups in the control breeding design used for this experimental study. The numbers in columns with markers refer to their length expressed in base pairs; the absence of a number in this column indicates the lack of a given genome copy. Detailed information is given in a footnote to the table.

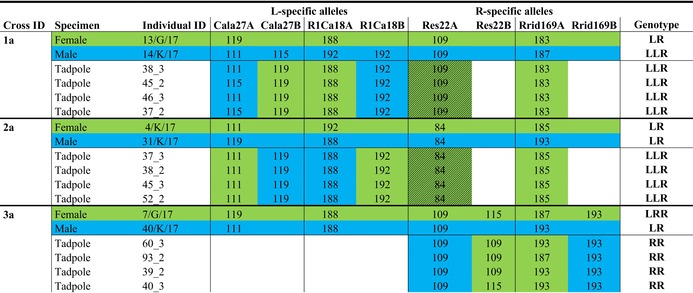

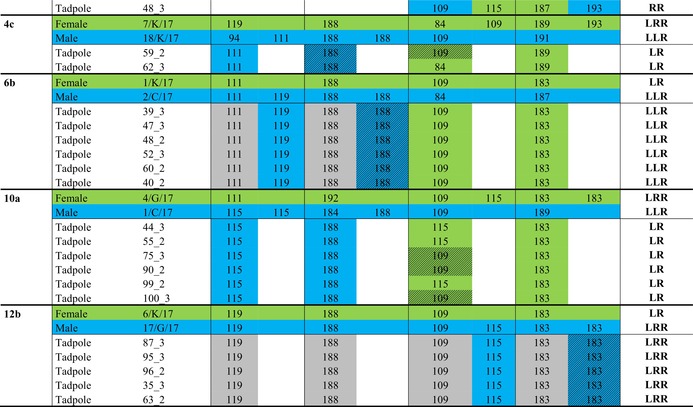

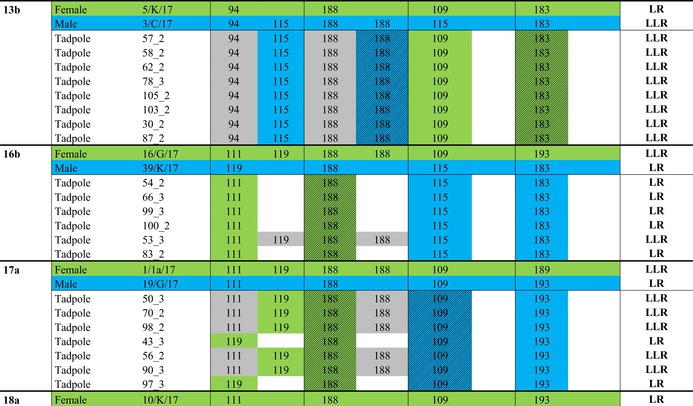

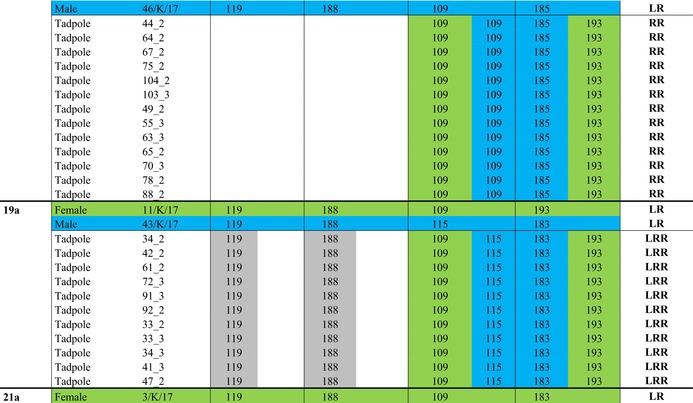

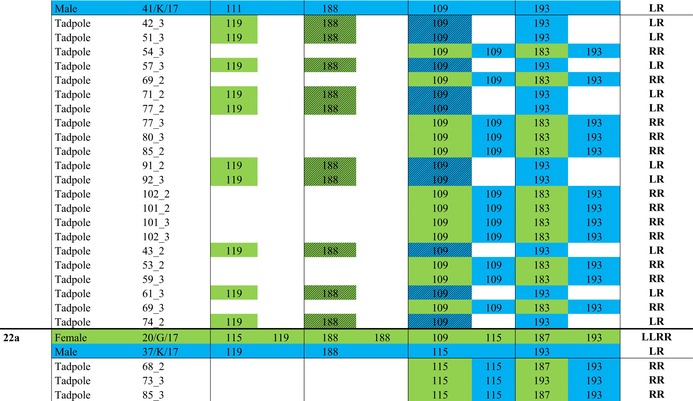

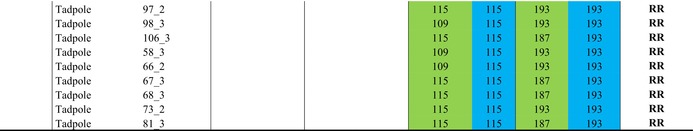

*Note:* Genotype inference based on L‐ and R‐specific markers: LL, alleles only at L‐specific loci (Cala27 and RlCA18) and lack of PCR products at R‐specific loci; LLR, one allele at both R‐specific loci and two alleles at least at one L‐specific locus; LLRR, two alleles at least at one L‐specific locus and two alleles at least at one R‐specific locus; LR, one allele at each of the four loci; LRR, one allele at both L‐specific loci and two alleles at least at one R‐specific locus; RR, alleles only at R‐specific loci (Res22 and Rrid169A) and lack of PCR products at L‐specific loci. Colors: Green, female genotypes and maternal alleles in tadpole genotypes (they can only come from the mother because they are present in her genome and not in the father's genome); Blue, male genotypes and paternal alleles in tadpole genotypes (they can only come from the father because they are present in his genome and not in the mother's genome); Gray, alleles of unknown origin that may come from either female or male genotype because they appear in the genomes of both; Hatched field alleles for which the origin (maternal: green‐hatched field or paternal: blue‐hatched field) was determined using a second marker from a pair of genome‐specific loci, for example, in cross no. 1a, all tadpoles in the Rrid169 locus (R‐specific) had Allele 183, which was present only in the mother's genome; hence it is known that she transfered “R” genome, and thus Allele 109 in the second R‐specific marker, that is, Res22, even though it was present in both the male and the female genomes, it must also have come from the mother's genome.
